# Alveolar epithelial regeneration in the aging lung

**DOI:** 10.1172/JCI170504

**Published:** 2023-10-16

**Authors:** SeungHye Han, G.R. Scott Budinger, Cara J. Gottardi

**Affiliations:** 1 Division of Pulmonary and Critical Care Medicine, Department of Medicine, and; 2 Cell and Developmental Biology, Northwestern University, Chicago, Illinois, USA.

## Abstract

Advancing age is the most important risk factor for the development of and mortality from acute and chronic lung diseases, including pneumonia, chronic obstructive pulmonary disease, and pulmonary fibrosis. This risk was manifest during the COVID-19 pandemic, when elderly people were disproportionately affected and died from SARS-CoV-2 pneumonia. However, the recent pandemic also provided lessons on lung resilience. An overwhelming majority of patients with SARS-CoV-2 pneumonia, even those with severe disease, recovered with near-complete restoration of lung architecture and function. These observations are inconsistent with historic views of the lung as a terminally differentiated organ incapable of regeneration. Here, we review emerging hypotheses that explain how the lung repairs itself after injury and why these mechanisms of lung repair fail in some individuals, particularly the elderly.

## Introduction

The lung epithelium is susceptible to viral, bacterial, and toxic insults, but this susceptibility is offset by a remarkable capacity to repair and regain function after injury. This regenerative capacity of the lung was seen recently in the majority of patients recovering from severe SARS-CoV-2 pneumonia (1). However, it declines with age, putting elderly people at disproportionate risk for mortality from acute and chronic lung diseases (2), as evidenced by the disproportionate impact of the COVID-19 pandemic on elderly populations (3). The age-related decline of lung repair in response to viruses and toxins that is observed in humans is in part recapitulated in murine models, which has enabled causal genetic studies to elucidate specific mechanisms underlying the decreased repair in older individuals (4–6). Furthermore, the development of innovative experimental techniques such as single-cell RNA sequencing analysis, in vivo lineage tracing technology, and 3D organoid culture systems has provided tools to probe region-specific epithelial stem or progenitor cell populations throughout the airways and alveoli (7–9). Here, we focus on our emerging understanding of alveolar epithelial repair mechanisms and hypotheses for their decline with age. We particularly focus on newly described epithelial populations identified by single-cell RNA sequencing in the alveolar space of humans and mice, and the small conducting airways including terminal and respiratory bronchioles of humans (10–20).

## Development and maintenance of the alveolar epithelium

The primary physiologic function of the mammalian lung is to supply oxygen sufficient to meet the metabolic needs of the organism and to remove carbon dioxide to prevent toxicity (21). This is accomplished by diffusion of these gases across a basement membrane shared by alveolar epithelial type I (AT1) cells and a specialized population of endothelial cells marked by expression of carbonic anhydrase 4 (*CAR4*) (22). AT1 cells are extremely large and thin cells that in the mouse can be conceptualized as bedsheets draped over approximately three alveoli (23, 24). AT1 cells represent approximately 50% of the cells in the alveolar epithelium but comprise 95% of the alveolar surface area (25). In stark contrast, alveolar epithelial type II (AT2) cells are relatively small cuboidal cells that secrete lipoproteins (surfactants) necessary to maintain alveolar surface tension and prevent regional alveolar atelectasis, and play key roles in the host response to infection (26, 27). Several fibroblast populations are found in the alveolar interstitium, and alveolar macrophages reside both in the alveolar luminal space and around bronchovascular bundles (28–30).

Repair of the alveolar epithelium after injury may involve the activation of developmental pathways, which might decline with advancing age. Development of the alveolar airspaces (i.e., alveolarization) starts postnatally during the alveolar stage and continues for 4 to 5 weeks in mice and at least 3 years in humans (31). During homeostasis, the turnover of AT2 cells in the alveolar space is thought to be slow, with some estimates suggesting a turnover of approximately 7% of murine alveoli per year (32). The normal doubling time of murine AT2 cells is long, ranging from months to more than one year, likely depending on age, anatomical location, or injury exposure (33, 34). Turnover of the lung extracellular matrix (ECM) is also thought to be slow. For example, deletion of *Lama3*, one of the matrix component–encoding genes secreted by epithelial cells, in mouse distal lung epithelium resulted in just an approximately 50% loss of the protein at 3 months (35). Whether these turnover rates slow with aging in either mice or humans represents an interesting but unresolved question, particularly given that pulmonary fibrosis, a disease of excessive ECM accumulation, is associated with advanced age and may be worsened by reduced rates of ECM turnover (36).

In normal young adult mice, lineage tracing methods revealed that AT2 cells both self-renew and give rise to AT1 cells, and thus serve as the primary progenitor cells responsible for alveolar epithelial maintenance (32, 34). Evidence suggests that only a subpopulation of AT2 cells (1%–20%) are poised to regenerate AT2 cells and form new AT1 cells, and this poised state is controlled by localized activation of β-catenin (33, 37). These Wnt-activated AT2 cells, lineage-labeled with the universal β-catenin target gene *Axin2*, manifest key features of stem cells: they are long-lived, with a greater capacity for cell division than *Axin2*-negative AT2 cells; they also show an ability to clonally expand (self-renew) and give rise to AT1 daughters. What controls the range in percentage of *Axin2*^+^ AT2 cells between distinct mouse *Cre* lines remains unknown. Using genetic tools to force-activate or inhibit β-catenin signaling in AT2 cells, Nabhan et al. affirmed that β-catenin signaling maintained the AT2 progenitor cell state without differentiation, whereas removal of β-catenin promoted the AT2-to-AT1 differentiation (33). How β-catenin–activated, *Axin2*-expressing AT2 cells are spatially maintained or inhibited within the narrow confines of AT2 cell niches is not fully understood. While the *Axin2*^+^ status of AT2 cells requires Wnt secretion from adjacent stromal cells (33), whether particular Wnts are spatially arranged to direct these decisions is under investigation (38). In addition, while AT1 renewal by AT2 progenitor cells appears biased toward perivascular and sub-mesothelial regions (32), *Axin2^+^* AT2 cells appear sporadically distributed throughout the lung (33, 37), suggesting that cues for these regional expansion zones are likely codirected by signals other than Wnts.

## Alveolar epithelium is susceptible to environmental injury

The alveolar epithelium has a large surface area (~70 m^2^ in humans) and is directly exposed to the ambient air, making it susceptible to airborne pathogens, particulates, and toxins (25, 39, 40). The alveolar epithelium is protected by a thin layer of fluid rich in antimicrobial peptides whose viscosity and mucous content are precisely controlled (41–43). The epithelial lining fluid is continuously surveilled by alveolar macrophages (44, 45). Alveolar macrophages take up particles and toxins that are trapped in the epithelial lining fluid, after which they detach from the alveolar epithelium and undergo mucociliary clearance to the pharynx, where they are swallowed to be excreted in the feces (43, 46). Age-related changes in the composition of alveolar lining fluid and in the function of alveolar macrophages might enhance the susceptibility to infections, particulate matter air pollutants, and smoking (47, 48), and contribute to the prevalence of alveolar diseases in the elderly (2). Damage to the alveolar epithelium and endothelium and lung inflammation contribute to the adverse health consequences of exposure to inhaled particulate matter air pollutants and smoking (47, 48). These include pneumonia, the most common cause of death from infectious diseases (49); chronic obstructive pulmonary disease (COPD), among the top four causes of death in the United States (49); lung cancer, the most common cause of cancer-related death in both men and women (50); and pulmonary fibrosis (51).

## Repair of the damaged alveolar epithelium

**Figure 1:**
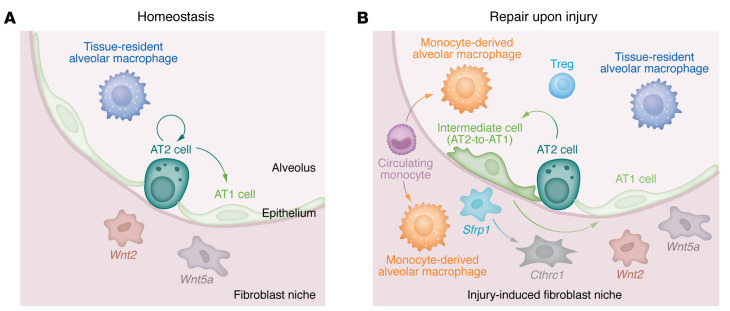
Repair of the alveolar epithelium after lung injury. (**A**) Alveolar epithelial type II (AT2) cells are small cuboidal cells that serve as a partially committed progenitor population. AT2 cells are required for the differentiation and maintenance of tissue-resident alveolar macrophages and are maintained by signals from adjacent mesenchymal cells, including Wnt2^+^ fibroblasts. Alveolar epithelial type I (AT1) cells are large flat cells that can spread over more than one alveolus. Their differentiation and maintenance in the niche also require signals from the adjacent mesenchyme, including Wnt5a^+^ fibroblasts. (**B**) In response to injury, AT2 cells undergo asymmetric division, with the smaller daughter cell regenerating the AT2 cell and the larger daughter cell differentiating into an AT1 cell. Single-cell RNA sequencing of the lung in murine models of injury, repair, and fibrosis and patients with pulmonary fibrosis identified a population of transitional cells with intermediate phenotypes between AT1 and AT2 cells that accumulate in areas of fibrosis. Transitional cells and profibrotic monocyte-derived alveolar macrophages recruited in response to epithelial injury drive the differentiation of fibroblasts into an intermediate phenotype characterized by expression of *Sfrp1* and subsequently a myofibroblast, characterized by *Cthrc1*. Tregs provide signals that directly or indirectly enhance epithelial repair.

Studies in mouse models of lung injury and repair combined with studies of murine and human alveolar epithelial cells in organoid cultures have elucidated pathways of alveolar repair in some detail, and the reader is referred to comprehensive reviews on this topic (8, 52) (Figure 1). Nabhan et al. showed that while the AT2 progenitor phenotype is locally maintained by Wnt-producing niche cells (33), AT2 injury appears to disrupt this exclusive reliance on niche cells for Wnt ligands, and upregulates an entire suite of Wnts across the bulk population of AT2 cells, presumably to recruit a greater proportion of the AT2 cell population for expansion and differentiation to AT1 cells (33). These data are consistent with evidence that mouse (33) and human (37) AT2 cells in organoid culture proliferate in response to Wnt/β-catenin pathway activation. The notion that normal alveolar epithelial turnover is controlled by narrowly confined signals between epithelial and niche cell interactions, and that injury or advancing age blurs and disrupts these signals, is a powerful concept that may inform our understanding of the age-related susceptibility to alveolar disease.

By applying single-cell RNA sequencing analysis to lung explants from patients undergoing lung transplantation for pulmonary fibrosis and lung tissue from decedents without lung disease, several groups identified a population of epithelial cells that simultaneously express genes that are normally restricted to either AT2 or AT1 cells (15, 16, 18). These cells also express high levels of cytokeratins, genes associated with cell cycle arrest (*CDKN2A*, *CDKN1A*), and genes sometimes associated with senescence (*SERPINE1*). In parallel, single-cell RNA sequencing of murine models of lung injury and fibrosis has identified a transcriptionally similar population of cells (10–13). These cells are variously referred to as Krt8^+^ alveolar differentiation intermediate (ADI) (12), damage-associated transition progenitors (DATPs) (13), and pre–alveolar type 1 transitional cell state (PATS) in mice (10), and KRT17^+^KRT5^lo^ (15) or aberrant basaloid cells (16) in humans. Using organoid models, Tata and colleagues (10) demonstrated that these cells represent a transitional cell population that accumulates during the differentiation of AT2 to AT1 cells, a finding confirmed by other groups in both murine and human systems (10–13, 15, 16, 18). We found these cells accumulated in the lungs of patients dying from or requiring lung transplantation after COVID-19 (18), a finding subsequently confirmed by others (53, 54). In a single-cell atlas of murine lung development, Sucre and colleagues identified a transcriptionally similar population of cells that emerges during postnatal alveologenesis (14). It is worth noting that selective killing of AT2 cells in mice can be restored by a temporally distinct, two-step process: AT2 death is first accompanied by AT1 cell membrane expansion over the original AT2 cell footprint; secondarily, small airway cells migrate, reprogram, and replace AT2 cells in the distal lung (55). While the accumulation of transitional cells is clearly associated with lung pathology, whether these cells are pathogenic or merely markers of ongoing alveolar differentiation is still unresolved. Furthermore, it is not clear whether the same injury results in a greater accumulation of transitional cells in old compared with young animals.

## Progenitors in human respiratory bronchioles

In the distal lung of mice and rats, there is an abrupt transition between tubular airway structures, lined by cuboidal epithelium, and the alveolar epithelium discussed above (56). However, in humans, nonhuman primates, ferrets, and other mammals, there is an additional anatomic structure called the respiratory bronchiole, where tubules lined by cuboidal epithelium are intermittently interrupted by alveolar epithelium, creating a small airway that participates in gas exchange (19, 20, 39). Respiratory bronchioles are a primary site of many age-related human pathologies, including emphysema (an important pathologic finding in patients with COPD) (20) and bronchiolitis obliterans, seen in patients with chronic lung allograft dysfunction after lung transplantation (57), as a complication of some systemic autoimmune disorders (58), and in response to specific inhaled toxins (59, 60). Two groups independently applied single-cell RNA sequencing and single-molecule fluorescence in situ hybridization (sm-FISH) to microdissected regions of the human lung and identified a transcriptomically novel progenitor cell population in the respiratory bronchioles (19, 20). Using organoid cultures and computational models to infer the origins and progeny of these progenitor cells, Murthy et al. suggested that these “AT0” cells (marked by expression of *SCGB3A2*, *SFTPC*, and *AGER*) originate *from* AT2 cells, and can give rise to either AT1 cells or cuboidal terminal bronchiole secretory cells (marked by expression of *SCGB3A2* and *SFTPB*) (19). In contrast, Basil et al. suggested that respiratory airway secretory cells (also marked by expression of *SCGB3A2*) undergo unidirectional differentiation *into* AT2 cells (20). Further studies will be required to resolve the fate of these cells during lung injury and repair, and the functional significance of these cells in the development of lung diseases.

## Other progenitor populations implicated in age-related lung disease

Influenza A infection in mice results in diffuse lung injury that is heterogeneous in severity across lung regions. Kumar et al. noted that in areas of severe injury, damaged alveolar epithelium was abnormally replaced by “pods” of cuboidal epithelial cells marked by expression of KRT5 and p63, which are typically expressed in airway epithelia (61). Independent groups identified these cells as originating from the distal airway (37, 61–63), and this heterogeneous cell population appears to migrate to severely damaged areas and undergo expansion (64). Inhibition of Notch signaling via HIF-1α deletion or enhanced Wnt/β-catenin activity promotes the differentiation of this cell population into AT2/AT1 cells, rather than KRT5/p63^+^ (64, 65). The resulting KRT5/p63^+^ pods persist over time (66), and this response seems adaptive, as ablation of KRT5^+^ cells during influenza A–induced injury led to the accumulation of inflammatory cells and the development of fibrosis (67). Homologous structures have been observed in humans with idiopathic pulmonary fibrosis, and their ontologic origins are under investigation (68).

Bronchioalveolar stem cells are a rare cell population, coexpressing the club cell marker CC10 (SCGB1A1) and the AT2 marker SP-C (69). These cells reside at the bronchoalveolar duct junction in mice, and can serve as an alternate progenitor cell population in some conditions (70–73). However, these cells have not been observed in humans. Some speculate that their function is performed by progenitor cells in the respiratory bronchioles in humans (19, 20).

In addition, it has been suggested that a small population of AT1 cells may “reverse-differentiate” back to an AT2 fate after injury through alterations in actin organization that promote nuclear accumulation of the Hippo pathway transcription factors YAP and TAZ (74, 75). Thus, as with other regenerative organ systems (e.g., gut), a variety of differentiated lung epithelial cells show capacity to reprogram and replenish proximal cell types through iterative bidirectional communication between epithelia and stroma components. Indeed, understanding the molecular rules of reparative versus pathogenic epithelial-mesenchymal cross-communication and how they change with advancing age may guide therapeutic approaches to amplify or attenuate these circuits.

## The aging mesenchyme’s role in alveolar epithelial plasticity

The epithelium and mesenchyme form reciprocal interactions during homeostasis, which are likely critical for stem cell maintenance that involves signaling through a variety of growth and differentiation factors. Gokey et al. showed that treatment of fibroblasts isolated from old mice with retinoic acid improved the age-related loss of alveolar epithelial differentiation in an organoid model, which they attributed to enhanced expression of platelet-derived growth factor subunit A (PDGFA) (76). Nabhan et al. found that inhibiting Wnt secretion from PDGF receptor A–positive (PDGFRA^+^) fibroblasts (via removal of the Wnt chaperone *Wntless*) reduced the number of *Axin2*^+^ AT2 cells (33). Consistent with a model in which Wnt signals from fibroblasts maintain an epithelial stem cell niche, Lee et al. identified a population of alveolar mesenchymal cells marked by expression of the stem cell marker leucine-rich repeat–containing G protein–coupled receptor 5 (LGR5), which also expressed high levels of Wnt ligands (77). Similarly, Murthy et al. identified a population of LGR5-expressing fibroblasts in human respiratory bronchioles, which they implicated in the development of the terminal bronchiole secretory cells and AT0 cells (19). Since LGR5 itself is a Wnt/β-catenin target gene (78), and Wnt/β-catenin–activated (Axin2^+^) mesenchymal cells enhance AT2 cell expansion and self-renewal (79), Wnt signals in both the epithelial and fibroblast niche cells appear to be critical for alveolar epithelial regeneration. Other pathways, including FGF signaling, have also been identified as regulating the interaction between epithelium and mesenchyme for stem cell maintenance (79–81).

Recently, Mayr et al. applied serial single-cell RNA sequencing and sm-FISH to the mouse model of bleomycin-induced lung injury and fibrosis (82), and identified a fibroblast population characterized by expression of *Sfrp1* in peribronchiolar, adventitial, and alveolar locations. *Sfrp1*^+^ fibroblasts appear to precede the emergence of *Cthrc1*-expressing myofibroblasts (82). *Sfrp1* is expressed in lung mesenchyme during embryogenesis (83), but the expression level appears to be decreased after birth (84). We also observed the expansion of a transcriptionally similar population of *Sfrp1*^+^ fibroblasts in a murine model of lung epithelial deficiency of mitochondrial electron transport chain (ETC) complex I, which results in the accumulation of abundant transitional epithelial cells in alveoli (85). In humans, a population of fibroblasts characterized by expression of *CTHRC1* and *TGFB1* was identified in lung explants from patients with idiopathic pulmonary fibrosis (86). These fibroblasts drove the differentiation of human AT2 cell ex vivo organoid cultures toward a basal cell phenotype reminiscent of transitional cells (68). Notably, stromal cell aging can negatively impact AT2 progenitor activity in organoid growth assays, but the soluble niche factors responsible for this deficit remain unclear (87). Collectively, these results suggest bidirectional signals between epithelium and mesenchyme that are important during the homeostatic response to injury.

## Age-related decline in alveolar regeneration in murine models

Epidemiologic data consistently implicate age as the most important risk factor for the development of the most prevalent acute and chronic lung diseases, including pneumonia, COPD, lung cancer, and pulmonary fibrosis (2). This impact of age on the severity of lung disease is recapitulated in murine models. For example, compared with young adult mice, old mice develop more severe and persistent fibrosis in response to bleomycin, suffer higher mortality after influenza infection, and fail to completely repair the lung after influenza-induced lung injury (4, 5, 88, 89). Young adult mice can generate new alveoli after unilateral pneumonectomy, but this ability is lost in older mice, perhaps as early as 9 months of age (90, 91). Isolated AT2 cells from old mice generate fewer and smaller alveolar organoids when compared with those from young adult mice (92).

A combination of cell-autonomous and non-cell-autonomous mechanisms likely drives these age-related phenotypes. An example of a purely cell-autonomous mechanism of progenitor cell dysfunction is polyploidy, which offers cells a mechanism to become larger as cell size scales with DNA content (93). This is advantageous during injury when the barrier is leaky, but might come at a cost of reduced proliferative potential after subsequent injury (94). Polyploidy has been observed in differentiating AT2 cells in culture and in injured mice (95, 96), but its role in lung aging is still unexplored. An example of a purely non-cell-autonomous mechanism of age-related epithelial stem cell dysfunction can be found in the observation that Tregs from young but not old mice drive alveolar epithelial repair after adoptive transfer (88). In the remainder of this Review, we discuss both cell-autonomous and non-cell-autonomous changes in the aging lung that might impair epithelial repair after injury. Shortened telomeres represent a clinically important case in which alveolar epithelial regeneration might be impaired by both cell-autonomous and non-cell-autonomous mechanisms (97, 98).

## Cellular stress response and aging

**Figure 2:**
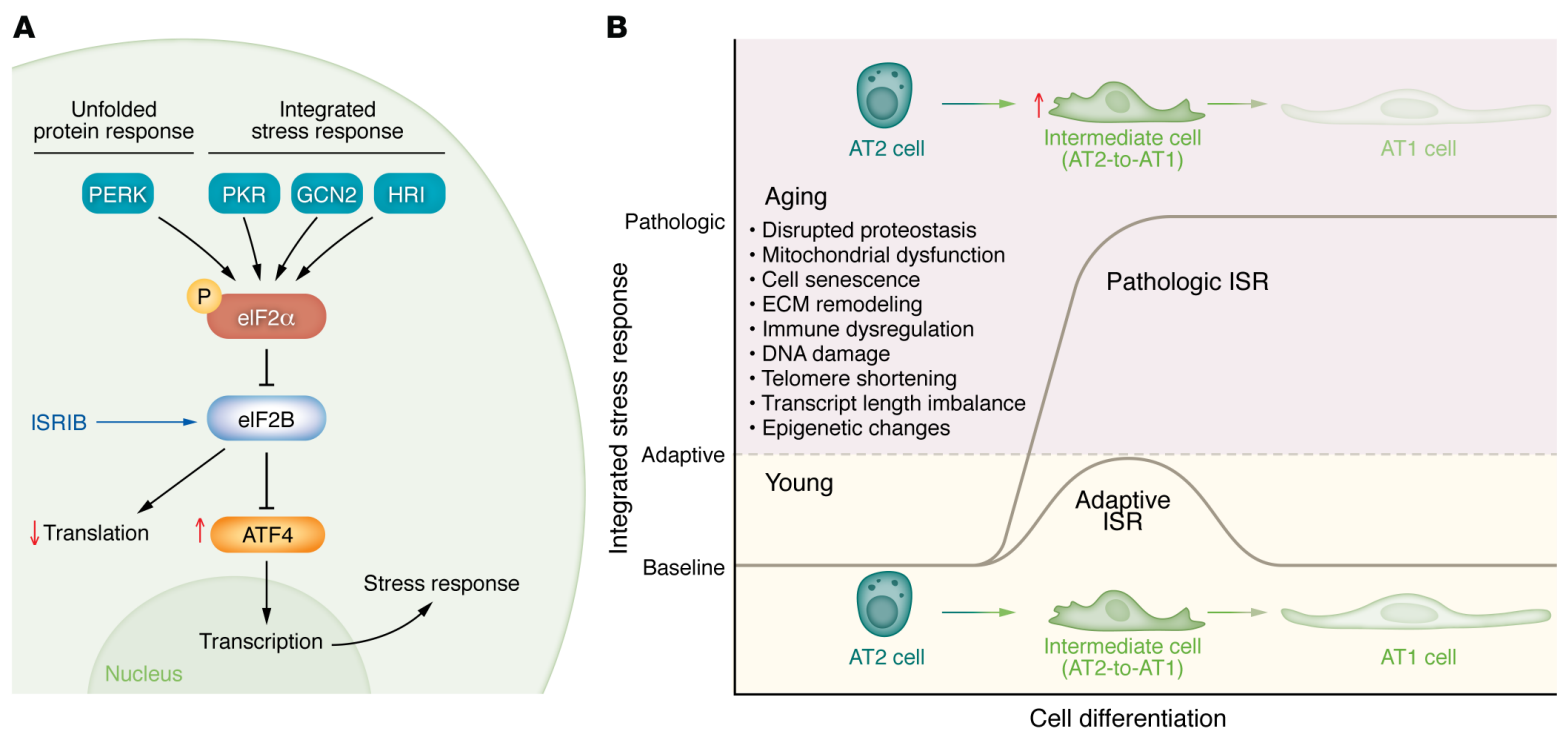
The integrated stress response and cell differentiation. (**A**) Integrated stress response (ISR) signaling. ISR activation inhibits global protein synthesis but induces ATF4 translation, both of which are improved by the ISR inhibitor (ISRIB). (**B**) Cell-autonomous and environmental signals during aging may contribute to activation of the ISR that serves as a barrier to AT2 differentiation. This could result in the accumulation of transitional cells that may preclude normal repair.

The lungs are constantly exposed to environmental stressors and infectious pathogens. Cellular stress is associated with chronic lung diseases and has been implicated in the pathogenesis of lung diseases such as COPD and pulmonary fibrosis. The integrated stress response (ISR) is an evolutionarily conserved mechanism that protects cells against a variety of stresses (reviewed in ref. 99). Although additional mechanisms exist to respond to these stresses, particularly proteostasis defects, we will focus our discussion on the ISR, as recent studies link it to several pathogenic processes, encouraging further consideration of its role in aging. ISR activation is induced by four specialized kinases (PERK, PKR, GCN2, and HRI), which sense and respond to distinct environmental stressors (Figure 2A). These kinases all phosphorylate eukaryotic translation initiation factor 2 subunit α (eIF2α) at Ser^51^ (100). Phosphorylation of eIF2α alters its binding to the guanine nucleotide exchange factor eIF2B, inhibiting its function, which results in a global inhibition of translation (101) but preferential translation of select mRNAs that harbor inhibitory upstream open reading frames in their 5′-untranslated regions, preventing translation during homeostasis (99, 102). These include ATF3, ATF4, ATF5, and CHOP (encoded by *Ddit3*) (103). These transcription factors, particularly ATF4, increase the expression of genes involved in stress response and one-carbon metabolism, and further enhance the expression of *Ddit3*. At the same time, ATF4 drives the transcription of *Gadd34*, which encodes a phosphatase that targets eIF2α, creating a negative-feedback loop that allows further translation. Consequently, ISR activation drives a transcriptional program that enables cells to restore cellular homeostasis or activate apoptosis during environmental stress. Evidence suggests that there is an age-dependent ISR induction in mice and *Caenorhabditis* *elegans* (104), but it remains unknown whether the age-dependent ISR induction is pathogenic or adaptive and what is the optimal level and duration of ISR activation. Notably, we have reported that pathologically high levels of ISR activation inhibit AT2-to-AT1 differentiation (85), and inhibiting ISR activation promotes AT2-to-AT1 differentiation and ameliorates bleomycin-induced lung fibrosis in mice (85, 105) (Figure 2B).

Endoplasmic reticulum (ER) stress has been linked to the development of fibrotic diseases, which is the subject of an excellent review in the *JCI* (106). Lawson et al. identified a mutation in *SFTPC* in a cohort of patients with familial pulmonary fibrosis (107). The mutation interferes with the normal folding of SFTPC in the ER, leading to the activation of ER stress pathways in alveolar epithelial cells (108). They went on to show that mice expressing this mutant protein, as well as mice treated with tunicamycin, showed increased fibrosis in response to bleomycin (109). Consistent with this hypothesis, Borok et al. found that mice deficient in *Grp78* (encoding BIP) were sensitized to bleomycin-induced fibrosis (110), and Katzen et al. showed that an inhibitor of ER stress attenuated fibrosis in mice expressing a human mutant SFTPC associated with familial pulmonary fibrosis (111). PERK is localized to the ER membrane and is activated upon ER stress, linking the ER stress pathways with the ISR, creating a challenge for understanding the respective roles of these pathways in the pathobiology of pulmonary fibrosis. In a screen for inhibitors of PERK, Sidrauski et al. identified a small-molecule inhibitor of the ISR (ISRIB) (99, 103), which was shown to exert beneficial effects across an array of developmental, neurodegenerative, and normal aging phenotypes in murine models (105, 112–121).

The laboratories of Peter Walter and David Ron used a structural approach to show that ISRIB interacts with eIF2B, restoring its guanine nucleotide exchange function even in the presence of phosphorylated eIF2B (99, 101, 103, 122) (Figure 2A). Because it facilitates the formation of eIF2B multimers, its effect is limited when ISR activation (evidenced by eIF2B Ser^51^ phosphorylation) is extremely robust, as occurs during viral infection (99). Hence, ISRIB functions as a tool compound, with a precisely defined molecular mechanism, that can dial back without completely inhibiting ISR signal. In murine models of bleomycin- and asbestos-induced lung fibrosis, our group found that ISRIB attenuated fibrosis severity in both young and old mice (105). We used a lineage tracing system to label AT2 cells and found that ISRIB increases the number of lineage-labeled AT1 cells and reduces the number of KRT8^+^ transitional cells both during bleomycin-induced fibrosis and in a murine model of adult alveologenesis after pneumonectomy. Collectively, these findings suggest a model in which persistent activation of the ISR stalls AT2-to-AT1 differentiation, preventing restoration of the alveolar epithelial barrier and promoting fibrosis (Figure 2B). Further support for this model was provided by Dobrinskikh et al., who found that ISRIB attenuated fibrosis in mice overexpressing *Muc5b* in the epithelium (119).

## Age-related mitochondrial dysfunction

Transcriptomic and metabolomic profiling of old animals, including humans, has revealed an age-related loss of mitochondrial transcripts, number, and function that varies between cell populations and tissues (123). Whether these changes are functionally important has been a subject of some debate, as maximal mitochondrial capacity typically far exceeds metabolic tissue demands even under stress conditions, and modest reductions in mitochondrial function induced by haploinsufficiency in mitochondrial genes do not accelerate aging phenotypes in unstressed animals (124). Interestingly, Liu and colleagues identified rare genetic variants in families with pulmonary fibrosis involved in the formation of mitochondrial ETC complex I (125), and Cuevas-Mora et al. identified alterations in mitochondrial function in alveolar epithelial cells with mutations in adaptor protein complex 3 β1 (Ap3b1), a cause of Hermansky-Pudlak syndrome (126). Several groups have shown that the ISR is activated in response to mitochondrial ETC dysfunction in vitro and in vivo (127–138). As noted above, we showed that deletion of mitochondrial ETC complex I from the alveolar epithelium during development led to accumulation of cells with transcriptional signatures similar to those of transitional cells during postnatal alveologenesis and models of lung injury, ultimately resulting in death of the animal (85). The accumulation of transitional cells was driven by an increase in the ratio of NADH to NAD^+^, which was necessary for activation of the ISR. Remarkably, inhibition of the ISR with ISRIB rescued the developmental phenotype in these mice, likely by inhibiting a pathway involving the mitochondrial proteins OMA1 and DELE1, which were identified by genetic screens as proteins linking mitochondrial inhibition with ISR activation (133, 134). These findings suggest the intriguing hypothesis that age-related mitochondrial dysfunction might drive excessive ISR activation during alveolar epithelial differentiation to preclude lung repair after injury (Figure 2B).

## Age-related senescence

Non-malignant cells maintained in culture eventually develop replicative senescence, characterized by stable expression of the cell cycle arrest proteins p16 and p21 (encoded by *CDKN2A* and *CDKN1A*, respectively) (139). These cells often increase in size, become resistant to apoptotic stimuli, and secrete cytokines, growth factors, and immune modulators, a condition referred to as the senescence-associated secretory phenotype (SASP) (140). Evidence for a role of senescence in age-related phenotypes comes from transgenic mouse models in which induction of the p16 promoter drives expression of a protein that causes apoptosis (141, 142). In these models, promoting apoptosis of p16-expressing cells resulted in improvements in age-related dysfunction (141, 143–146). Furthermore, improvements in age-related phenotypes are consistently observed when drugs that activate apoptosis or inhibit antiapoptotic proteins in p16-expressing cells, referred to collectively as senolytics, are administered to old mice (147–149). Based on these data, it is hypothesized that the accumulation of senescent cells within organs and tissues with advancing age results in high levels of SASP proteins that drive age-related phenotypes.

However, single-cell RNA sequencing atlases of the human and murine lung challenge this senescence hypothesis. First, *CDKN2A* and *CDKN1A* are expressed in multiple cell types in the normal lung, even during development, and the abundance of these cells is not expanded in normal aging (14, 150). Second, despite the ability to detect rare cell populations (as few as 0.01% of cells in the human lung cell atlas), single-cell RNA sequencing studies have not detected a population of transcriptionally distinct cells simultaneously expressing *CDKN2A* and SASP genes in the normal human lung (150), or in diseases like pulmonary fibrosis, where senescence has been suggested to play a role (15–17). It has been suggested that senescent cells may be selectively damaged or destroyed during tissue digestion for cell isolation, a possibility that should soon be addressed by spatial transcriptomic approaches. Alternatively, the beneficial effects of senolytics and therapies targeting p16-expressing cells on age-related outcomes may be attributable to the apoptosis of cells expressing p16 that are not senescent (151), or may result from a nonspecific response to the efferocytosis of apoptotic cells generated in response to senolysis, as has been suggested to explain the beneficial effects of mesenchymal stem cell administration (152, 153).

The transitional epithelial cells that emerge during pulmonary fibrosis described above express *CDKN2A*, *CDKN1A*, and other canonical genes associated with cellular senescence, but careful lineage tracing studies in mice and in human organoid systems suggest they are capable of differentiating into AT1 (10–13, 15, 18, 105). Given their high-level expression of *CDKN2A*, however, it is likely that these cells are targeted when apoptosis is induced in p16-expressing cells. The amelioration of fibrosis in response to these therapies indirectly supports the hypothesis that transitional cells are not merely markers of failed epithelial repair but are causally related to its pathobiology. In contrast, Negretti et al. observed these cells during normal mouse development (14), and we did not observe lung fibrosis when transitional cells were expanded in response to epithelial loss of mitochondrial ETC complex I (85).

All of the stem cell populations discussed above, including AT2 cells, are cuboidal cells that are extremely small in comparison with large, flat AT1 cells, which in mice can span more than one alveolus (23). As these stem cells differentiate, they must migrate to sites of injury and dramatically increase their membrane surface area either by spreading from a leading edge or by expanding in response to luminal fluid pressure (154). In this context, the finding that transitional epithelial cells upregulate genes encoding p53, p21, and p16, those associated with DNA damage signaling and cell cycle arrest, and genes involved in migration and matrix remodeling may reflect changes that facilitate these cellular processes rather than end-stage pathology (10–13). Indeed, a form of epithelial cell migration commandeered by “leader cell” behavior relies on p53/p21 signaling (155).

## Age-related changes in the ECM

There is emerging evidence that the ECM undergoes extensive remodeling and increases tissue stiffness during aging (36, 156). Similarly, several studies have reported age-related ECM changes in the lungs altering lung mechanics and structure (157). Atomic force microscopy has been used to demonstrate age-related increases in stiffness in the parenchymal and vessel compartments of the human lungs (158). Integrated analysis of transcriptomic and proteomic data from aged mice revealed ECM changes, including increased collagen IV and XVI and decreased Fraser syndrome complex proteins and collagen XIV (159). Also, the expression and solubility of fibrillar collagens are decreased in the lungs of aged mice (160). ECM alterations in the lungs are associated with chronic lung disease and have emerged as a potentially important player during lung development, homeostasis, and repair after injury (161). Recent studies suggest that changes in lung mechanical tension may be a cue for AT2 cells to proliferate and differentiate in order to repair damaged AT1 cells or damaged whole alveoli (162, 163). YAP is a transcription coactivator and acts as a stiffness sensor, regulating mechanotransduction. Using conditional knockout mice and pharmacologic tools, Liu et al. demonstrated that YAP in AT2 cells is necessary for post-pneumonectomy alveolar growth, which was regulated by JNK and p38 MAPK signaling upstream (162). They showed that Cdc42 was required for the activation of JNK, p38, and subsequently YAP in post-pneumonectomy alveolar growth. Furthermore, the loss of Cdc42 in AT2 cells induced spontaneous progressive lung fibrosis in aged mice, and led to impaired alveolar growth and fibrosis after pneumonectomy (163). Whether and how age-related ECM changes in the lung alter epithelial repair remains an important area for further investigation.

## Age-related immune dysregulation

A substantial body of literature points to dysregulation of the immune system with advancing age. This is characterized by systemic inflammation: increased levels of cytokines induced upon activation of the NLRP3 inflammasome (IL-1β and IL-18) in parallel with increased levels of TNF-α and IL-6 (164, 165). At the same time, adaptive immune responses and vaccine efficacy are also reduced in the elderly. Collectively, these changes might contribute to the enhanced susceptibility of the elderly to pathogens and result in hyperinflammatory responses during sterile and infectious injury (166). The molecular drivers of these changes are incompletely understood.

In parallel, immune-mediated repair of the lung declines with age. For example, Tregs contribute to lung repair after injury, but Tregs from old mice show a cell-autonomous loss of reparative function (88, 167). Alveolar macrophages have also been implicated in lung repair after injury (30). We observed an age-associated reduction in expression of cell cycle genes in alveolar macrophages in mice, accompanied by reduced numbers of alveolar macrophages (4). In contrast to Tregs, however, these changes were not cell autonomous, and instead were driven by the alveolar microenvironment. Interestingly, we did not observe changes in the levels of GM-CSF expressed by AT2 cells or the levels of GM-CSF in alveolar fluid with age. Instead, a transcriptomic analysis and in vitro studies implicated ECM changes, including levels of the major extracellular polysaccharide hyaluronan (4). The mechanisms by which immune cells interact with the epithelium or other lung cells to promote tissue repair and how these functions change with aging are poorly understood.

## Conclusions

Notably, while here we have focused on new insights into age-related changes that contribute to failed alveolar epithelial repair in the elderly, there are other mechanisms involved that we have not discussed for the sake of brevity. These include the accumulation of DNA damage with age (168), the loss of transcriptional fidelity leading to a length imbalance favoring long transcripts in the transcriptome (169), and telomere shortening, the effects of which have been localized to the alveolar epithelium in animal models (97, 170).

With technical innovations such as single-cell analysis and genetic mouse models, we have made dramatic advances in the past decade toward understanding the mechanisms of alveolar repair. Nevertheless, research in this area has been limited in both humans and mice, albeit for different reasons. In humans, sample collection is biased toward sicker patients, who are typically older. In the absence of specific recruitment strategies, the relative undersampling of young individuals underpowers studies comparing biological responses in patients over the lifespan. In animals, meaningful inclusion of age as a biological variable in causal genetic models requires time (18–24 months is considered old in a C57BL/6 mouse) and incurs substantial costs, both of which can easily exceed a typical five-year research award. Studies of lung aging will therefore require a concerted commitment by investigators conducting clinical and basic translational research and by funding agencies. Arguably, these investments are worthwhile given the strength of the epidemiologic link between advanced age and acute and chronic lung disease and the impact of these diseases on public health.

## Version 1 10/16/2023

Electronic publication
